# Urban PM2.5 exacerbates allergic inflammation in the murine lung *via* a TLR2/TLR4/MyD88-signaling pathway

**DOI:** 10.1038/s41598-017-11471-y

**Published:** 2017-09-08

**Authors:** Miao He, Takamichi Ichinose, Yasuhiro Yoshida, Keiichi Arashidani, Seiichi Yoshida, Hirohisa Takano, Guifan Sun, Takayuki Shibamoto

**Affiliations:** 1 Department of Environmental Health, School of Public Health, China Medical University, Shenyang, 110122 China; 2 Department of Health Sciences, Oita University of Nursing and Health Sciences, Oita, 870-1201 Japan; 3 Department of Immunology and Parasitology, School of Medicine, University of Occupational and Environmental Health, Fukuoka, 807-8555 Japan; 4 Environmental Health Division, Department of Environmental Engineering, Graduate School of Engineering, Kyoto University, Kyoto, 615-8530 Japan; 5 Department of Environmental Toxicology, University of California, Davis, California, 95616 USA

## Abstract

Nevertheless its mechanism has not been well explained yet, PM2.5 is recognized to exacerbate asthma. In the present study, the roles of toll-like receptor (TLR) 2, TLR4 and MyD88, in exacerbation of allergen-induced lung eosinophilia caused by urban PM2.5 was investigated. TLR2-, TLR4-, MyD88-deficient and WT BALB/c mice were intratracheally challenged with PM2.5 +/− ovalbumin (OVA) four times at 2-week intervals. PM2.5 increased neutrophil numbers and KC in bronchoalveolar lavage fluid and caused slight peribronchiolar inflammation in WT mice. However, these changes were attenuated, but not completely suppressed in gene-deficient mice, especially in MyD88^−/−^ mice. In WT mice, PM2.5 + OVA exacerbated OVA-related lung eosinophilia. This exacerbation includes increase of IL-5, IL-13, eotaxin and MCP-3; infiltration of eosinophils into the airway submucosa; proliferation of goblet cells in the airway epithelium; and the production of antigen-specific IgE and IgG1 in serum. All these effects were stronger in TLR2^−/−^ mice than in TLR4^−/−^ mice. In MyD88^−/−^ mice, this pro-inflammatory mediator-inducing ability was considerably weak and lung pathology was negligible. These results suggest that urban PM2.5 may exacerbate allergic inflammation in the murine lung *via* a TLR2/TLR4/MyD88-signaling pathway. PM2.5-bound trace microbial elements, such as lipopolysaccharide may be a strong candidate for exacerbation of murine lung eosinophilia.

## Introduction

High concentrations of atmospheric particulate matter less than 2.5μm in diameter (PM2.5) are common in Asian countries, especially in some Chinese megacities, and various respiratory health problems have become coincident with this^1, 2^. High levels of PM2.5 have been associated with an increase in numbers of chronic obstructive pulmonary disease (COPD) and asthma patients in China and Taiwan^3^.

Asthma, especially in children, is a chronic disease with airway obstruction, and is characterized by chronic allergic inflammation of the airways with superimposed episodes of acute inflammation^4^. Acute exacerbations are the most clinically significant feature of asthma. They are represented by increase of distal eosinophilic airway inflammation associated with exaggeration of symptoms, such as cough, chest tightness and dyspnoea^5^. PM2.5 is known to be related to the development and exacerbation of asthma. For example, PM2.5 promotes sensitization to common aeroallergens and the development of allergic respiratory diseases^6^. Ambient PM2.5 levels show a positive correlation with upsurges in emergency department visits for asthma^7, 8^, hospital admissions for asthma in children^9, 10^, and worsening wheezing and dyspnoea^11^. Furthermore, asthma morbidity has been positively associated with daily ambient PM2.5 concentrations, in both warm and cool seasons^12^. The composition of PM2.5 may contribute to a higher prevalence and incidence of asthma^13, 14^.

Anthropogenic PM2.5 contains large amounts of toxic chemicals formed by the combustion of fossil fuel. Chemicals identified to date include polycyclic aromatic hydrocarbons (PAHs), sulfates (SO_4_^2−^)/nitrates (NO_3_^−^), transition metals (i.e. Fe, Cu, Ni), and microbial elements such as lipopolysaccharide (LPS) and β-glucan^15, 16^.

In our previous work using a murine asthma model, we have shown that urban dust collected from the air in Beijing, China exacerbated ovalbumin (OVA)-associated murine lung eosinophilia^17^, as did PM2.5 collected from the air in Shenyang, China^15, 16^ and PM2.5-rich dust collected from the air in Fukuoka, Japan^18^. However, the precise exacerbating factors contained within urban PM2.5 and the mechanisms of action are not fully understood. Therefore, studies to clarify the mechanisms involved in exacerbation of murine lung eosinophilia are necessary.

Toll-like receptors (TLRs) expressed by antigen presenting cells (macrophages, dendritic cells) and other various cells (e.g., airway epithelial cells) are innate immune sensors, which recognize microbial pathogen-associated molecular patterns (bacteria, fungi, and virus structures) as well as endogenous danger molecules from host cells. TLR2 is a receptor for β-glucans of fungi and peptidoglycans of Gram-positive bacteria^19^, while TLR4 detects LPS^20^. Myeloid differentiation factor 88 (MyD88) is a downstream signalling adapter protein and essential for cytokine production in response to TLR ligands^21^. We have recently reported that MyD88 downstream of TLR2 and TLR4 is a key protein in OVA-induced exacerbation of murine lung eosinophilia by Asian sand dust^22^.

Investigation of the role of TLR2, TLR4 and MyD88 in the exacerbation of allergen-induced lung eosinophilia caused by PM2.5 was conducted to clarify the relationship between microbial elements present in PM2.5 and disease exacerbation in a mouse model of asthma. Gene deficient (TLR2^−/−^, TLR4^−/−^ and MyD88^−/−^) and wild type (WT) BALB/c mice were intratracheally challenged with OVA and/or PM2.5 in the present study.

## Results

### Components in PM2.5 samples

PM2.5 includes constituents such as metal, sulfate, organic carbon (OC), and elemental carbon (EC); its chemical composition varies geographically depending on natural and/or anthropogenic generating sources. Table 1 shows the results of the major elements analysis. A total of 8 major elements were identified, along with OC and EC. In PM2.5, the concentration of K (18 µg/mg) was the highest, followed by Si (13 µg/mg), Fe (6.8 µg/mg), Zn (5.4 µg/mg), Na (4.7 µg/mg), Ca (4 µg/mg), Al (3.7 µg/mg), and Pb (1.7 µg/mg). OC and EC were 84 µg/mg and 182 µg/mg in PM2.5, respectively.

**Table 1:** Concentration of chemical elements, water soluble components, LPS and β-glucan in PM2.5.

Elements (µg/mg) Compositions		Trace (ng/mg) elements		Trace (ng/mg) elements		Water soluble (µg/mg) components		Microbial (pg/mg) elements	
Si	13	Sc	<9.1	Mo	110	SO_4_^2−^	160	LPS	83
Na	4.7	V	60	Sb	90	NO_3_^−^	130	β-glucan	410
Al	3.7	Cr	160	Cs	11	CL^−^	40		
K	18	Mn	510	Ba	71	Na^+^	4.6		
Ca	4	Ti	370	La	3.5	NH_4_^+^	110		
Fe	6.8	Co	8.5	Ce	5.7	K^+^	17		
Zn	5.4	Ni	72	Sm	<1.7	Mg^2+^	1.6		
Pb	1.7	Cu	340	Hf	0.3	Ca^2+^	2.9		
		As	380	W	12				
OC	84	Se	70	Th	<2.5				
EC	182	Rb	68	Cd	28				

OC: organic carbon; EC: element carbon.

Table 1 also shows the results of the minor elements analysis. Mn had the highest concentration (510 ng/mg), followed by As (380 ng/mg), Ti (370 ng/mg), Cu (340 ng/mg), Cr (160 ng/mg), Sb (90 ng/mg), Ni (72 ng/mg), Ba (71 ng/mg), Se (70 ng/mg), Rb (68 ng/mg), V (60 ng/mg), Cd (28 ng/mg), W (12 ng/mg), Cs (11 ng/mg), Co (8.5 ng/mg), Ce (5.7 ng/mg) and La (3.5 ng/mg).

The anion concentrations were SO_4_^2−^ (160 µg/mg), NO_3_^−^ (130 µg/mg), and Cl^−^ (40 µg/mg). The cation concentrations were NH_4_^+^ (110 µg/mg), K^+^ (17 µg/mg), Na^+^ (4.6 µg/mg), Mg^2+^ (1.6 µg/mg), and Ca^2+^ (2.9 µg/mg). The microbial levels of LPS and β-glucan were 83 pg/mg and 410 pg/mg, respectively.

Table 2 shows the results of the PAHs analysis. A total of 14 PAHs was identified. The concentration of BjF (448 µg/g) was the highest, followed by BeP (233 µg/g), DBA (217 µg/g), PYR (208 µg/g), FLU (153 µg/g) and BaA (108 µg/g). The concentration of the most potent carcinogen, BaP, was 61.1 µg/g.

**Table 2:** Concentration of PAHs in PM2.5.

PAHs	(μg/g)
Chrysene (CHR)	83.6
Fluorene (FLU)	153
Benzo[a]anthracene (BaA)	108
Pyrene (PYR)	208
Benzo[a]pyrene (BaP)	61.1
Benzo[k]fluoranthene (BkF)	26.7
Benzo[b]fluoranthene (BbF)	31.7
Dibenzo[a,h]anthracene (DBA)	217
Benzo[e]pyrene (BeP)	233
Benzo[j]fluoranthene (BjF)	448
Perylene	20.1
Benzo[g,h,i]perylene (BPE)	53.3
Indeno[1,2,3-cd]pyrene (IPR)	57.2
Coronene	24.2

PM2.5 and CPM samples were analyzed for PAHs by a HPLC equipped with a fluorescence detector and a 4.0 mmφ × 250 mm column a packed withWakosil-II 5 C 18HG.

### Inflammatory cells in BALF

**Figure 1:**
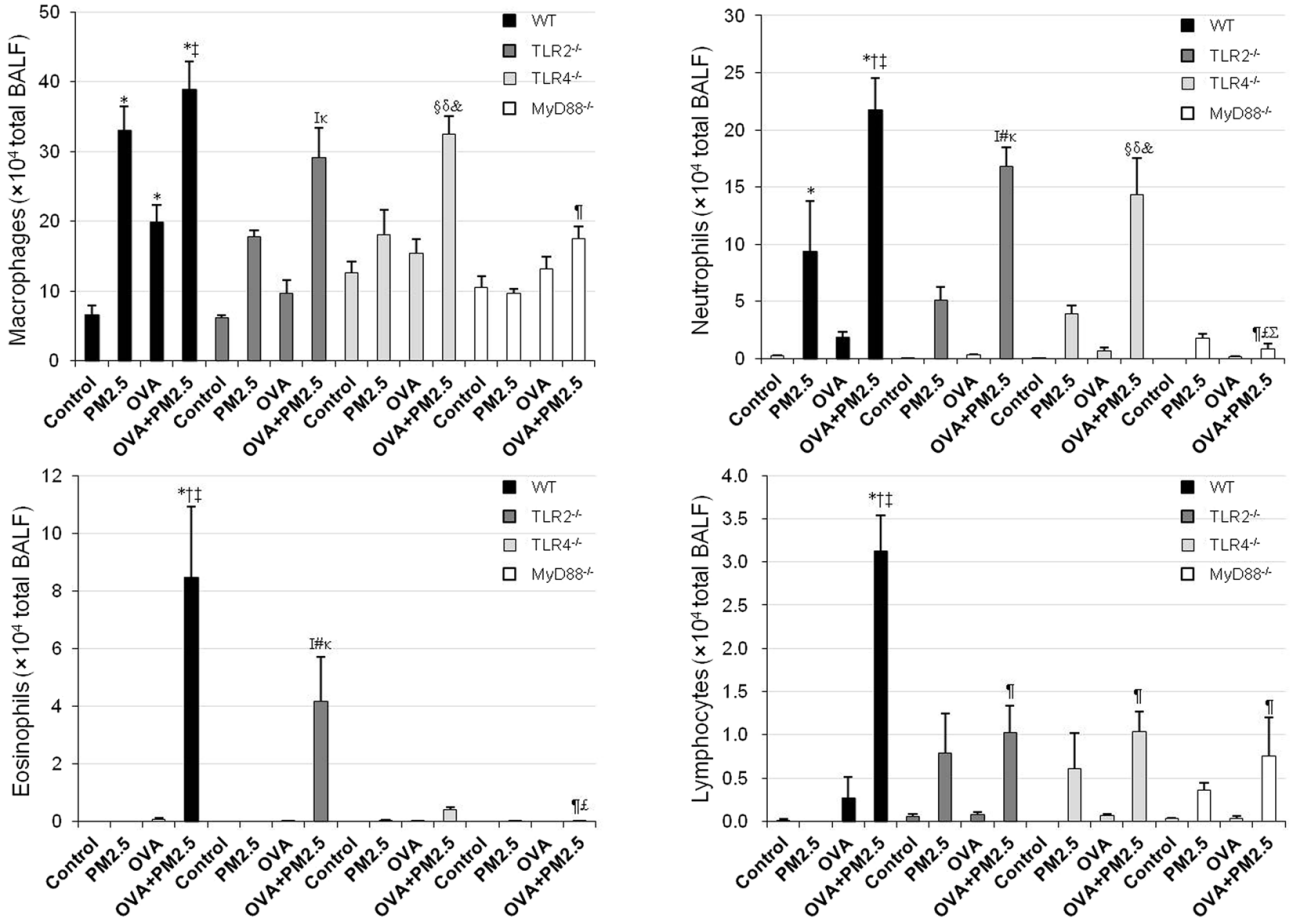
Inflammatory cells in BALF. Differential cell counts were assessed in cytologic preparations stained with Diff-Quik. All values are expressed as mean ± SE (n = 6). ^*^p < 0.05 vs. WT Control; ^†^p < 0.05 vs. WT PM2.5; ^‡^p < 0.05 vs. WT OVA; ^¶^p < 0.05 vs. WT OVA + PM2.5; ^I^p < 0.05 vs. TLR2^−/−^ Control; ^#^p < 0.05 vs. TLR2^−/−^ PM2.5; ^κ^p < 0.05 vs. TLR2^−/−^ OVA; ^£^p < 0.05 vs. TLR2^−/−^ OVA + PM2.5; ^§^p < 0.05 vs. TLR4^−/−^ Control; ^δ^p < 0.05 vs. TLR4^−/−^ PM2.5; ^&^p < 0.05 vs. TLR4^−/−^ OVA; ^Σ^p < 0.05 vs. TLR4^−/−^ OVA + PM2.5.

As shown in Fig. 1, PM2.5 significantly increased macrophages and neutrophils over the control in WT mice, but not significantly increased these cells in TLR2^−/−^, TLR4^−/−^ and MyD88^−/−^ mice. PM2.5 tended to increase lymphocytes in TLR2^−/−^, TLR4^−/−^ and MyD88^−/−^ mice. OVA + PM2.5 significantly increased neutrophils, eosinophils and lymphocytes compared to the controls, PM2.5− and OVA-only counterparts in WT mice, TLR2^−/−^ and TLR4^−/−^ mice, but not increased these inflammatory cells in MyD88^−/−^ mice. The eosinphil number in WT mice was greater than in TLR2^−/−^ mice, whereas it was lower in TLR4^−/−^ mice or undetectable level in MyD88−/− mice.

### Cytokine and chemokine levels in BALF

**Figure 2:**
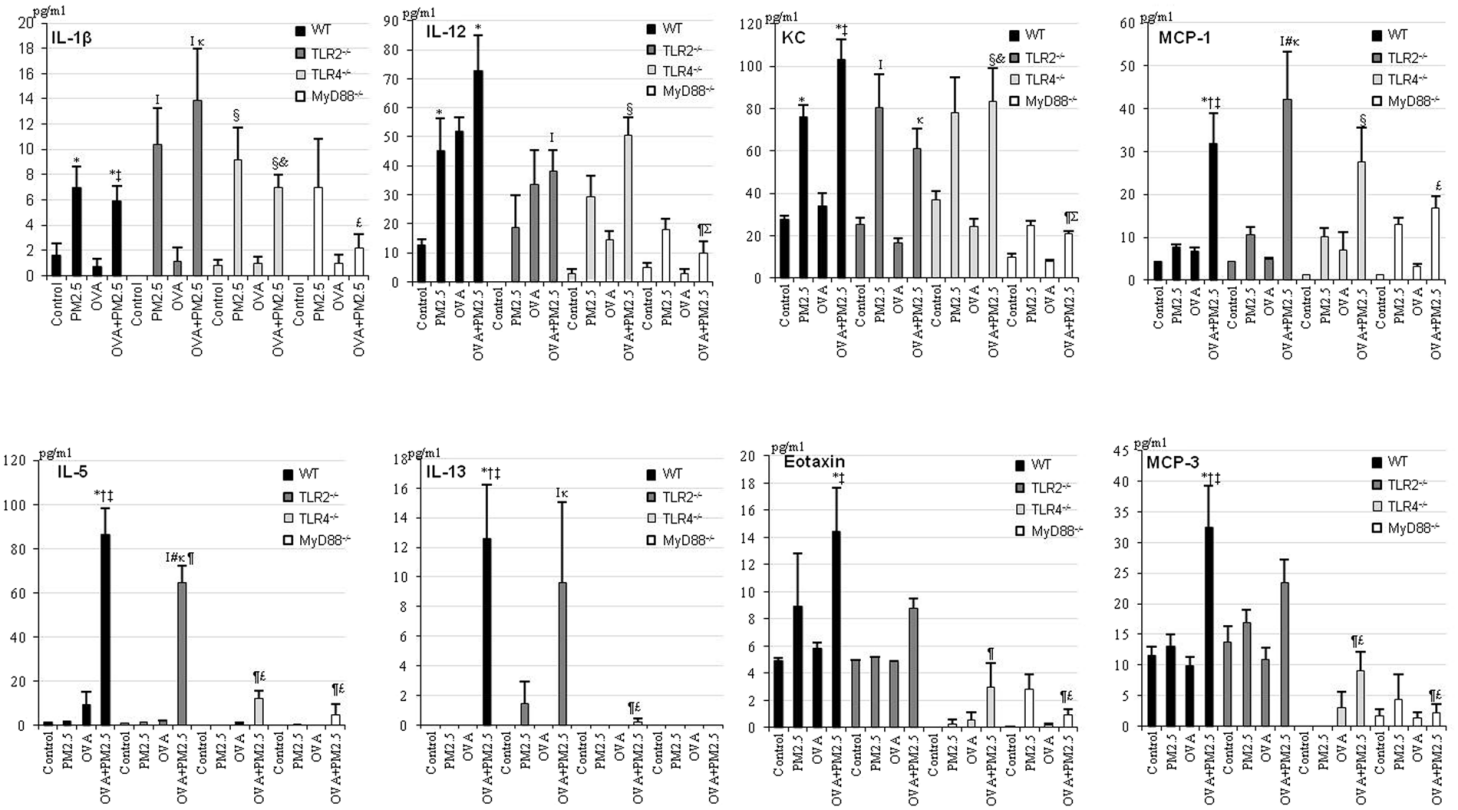
Expression of cytokines and chemokines in BALF. All values are expressed as mean ± SE (n = 6). *p < 0.05 vs. WT Control; ^†^p < 0.05 vs. WT PM2.5; ^‡^p < 0.05 vs. WT OVA; ^¶^p < 0.05 vs. WT OVA + PM2.5; ^I^p < 0.05 vs. TLR2^−/−^ Control; ^#^p < 0.05 vs. TLR2^−/−^ PM2.5; ^κ^p < 0.05 vs. TLR2^−/−^ OVA; ^£^p < 0.05 vs. TLR2^−/−^ OVA + PM2.5; ^§^p < 0.05 vs. TLR4^−/−^ Control; ^&^p < 0.05 vs. TLR4^−/−^ OVA; ^Σ^p < 0.05 vs. TLR4^−/−^ OVA + PM2.5.

As shown in Fig. 2, PM2.5 alone and OVA + PM2.5 significantly increased IL-1β in WT, TLR2^−/−^ and TLR4^−/−^ mice; KC in WT, TLR2^−/−^ and MyD88^−/−^ mice; and MCP-1 in MyD88^−/−^ mice over the controls. PM2.5 alone tended to increase IL-1β in MyD88^−/−^ mice and the increased level was same as that of WT mice.

OVA + PM2.5 significantly increased IL-12 in WT, TLR2^−/−^ and TLR4^−/−^ mice and MCP-1 in TLR2^−/−^ and TLR4^−/−^ mice relative to the controls. OVA + PM2.5 also significantly increased Th-2 cytokine IL-5 in WT, TLR2^−/−^ and TLR4^−/−^ mice, IL-13 in WT and TLR2^−/−^ mice, eosinophil-relevant MCP-3 in WT mice compared to the controls, PM2.5- and OVA-only counterpart and eotaxin in WT mice compared to the controls and OVA-only counterpart, but caused less or no secretion of these cytokine and chemokine in MyD88^−/−^ mice. Furthermore, an increase of the above cytokine and chemokine in TLR2^−/−^ mice were higher than those in TLR4^−/−^ mice.

### Pathologic changes in the airways

**Figure 3:**
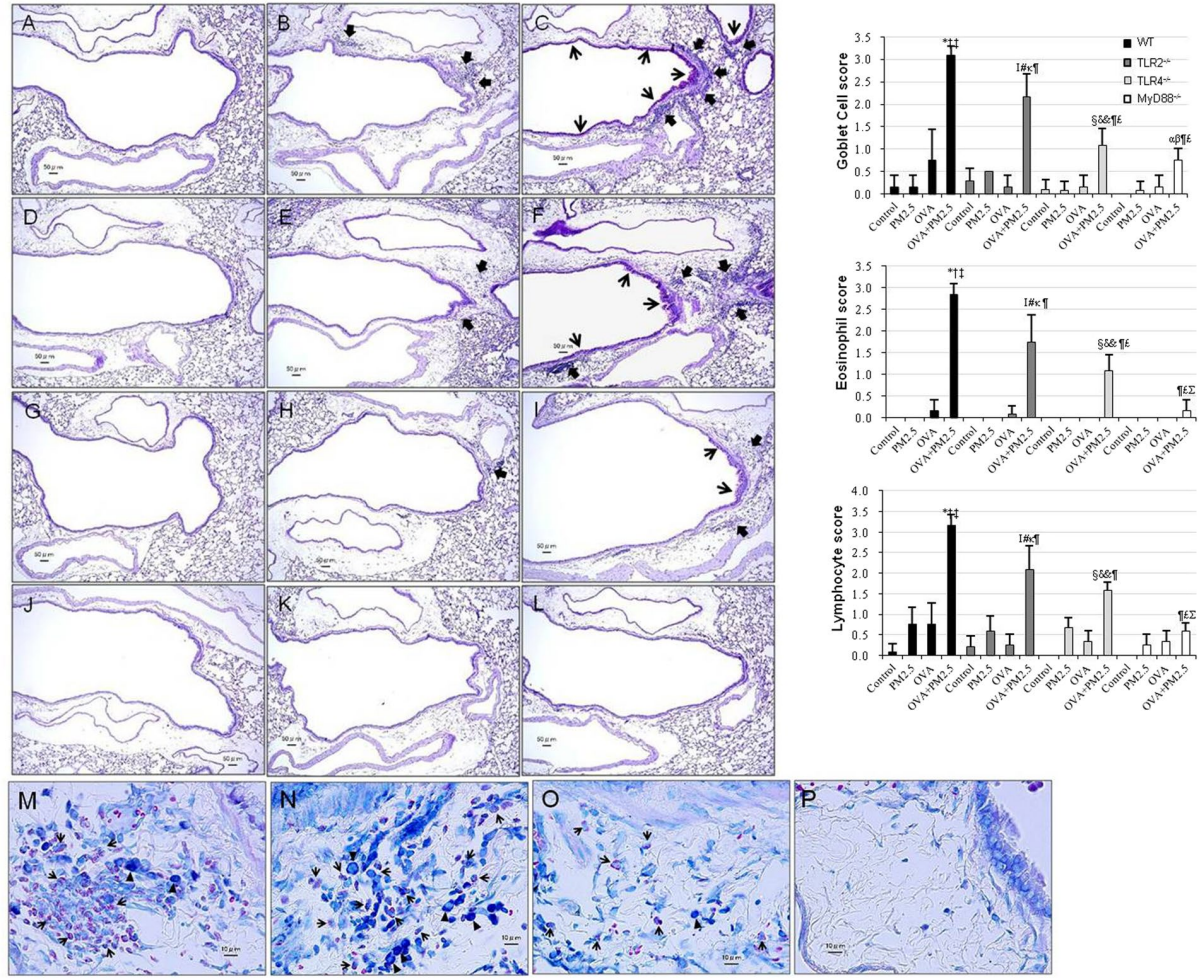
Pathological changes in mice lungs. (**A**) WT Control; (**D**) TLR2−/− Control; (**G**) TLR4−/− Control; (**J**) MyD88−/− Control: no pathological changes in lungs treated with saline. (**B**) WT PM2.5: very slight proliferation of airway epithelial cells, and peribronchiolar inflammation due to slight infiltration of inflammatory cells into the submucosa of airways. (**C**) WT OVA + PM2.5: moderate proliferation of goblet cells (thin arrow) that have mucus stained pink with PAS in the airway epithelium, and moderate to marked infiltration of inflammatory cells (arrow) into the submucosa of airways. Mild to moderate fibrous thickening of the subepithelial layer in the airway also was seen. (**E**) TLR2−/− PM2.5: proliferation of airway epithelial cells, peribronchiolar inflammation due to very slight infiltration of inflammatory cells (arrow) into the airway submucosa. (**F**) TLR2−/− OVA + PM2.5: mild to moderate proliferation of goblet cells (arrow) in the airway epithelium, inflammatory cells in the airway submucosa. (**H**) TLR4−/− PM2.5: no pathological changes in airway epithelial cells and very slight infiltration of inflammatory cells (arrow) into the airway submucosa. (**I**) TLR4−/− OVA + PM2.5: slight proliferation of goblet cells (thin arrow) in the airway epithelium, and slight infiltration of inflammatory cells (arrow) into the airway submucosa. (**K**) MyD88−/− PM2.5: no pathological changes in airway epithelial cells and no inflammatory cells in the airway submucosa. (**L**) MyD88−/− OVA + PM2.5: no significant pathological changes in airway epithelium or airway submucosa. (**A–L**) PAS stain; Bar = 50 μm. (**M**–**P**) Infiltration of inflammatory cells into the airway submucosa. May-giemsa stain; bar = 10 μm. (**M**) WT OVA + PM2.5: marked infiltration of eosinophils into the airway submucosa. (**N**) TLR2−/− OVA + PM2.5: moderate infiltration of eosinophils. (**O**) TLR4^−/−^ OVA + PM2.5: slight infiltration of eosinophils. (**P**) MyD88^−/−^ OVA + PM2.5: no significant pathological changes in the airway submucosa. Arrows show eosinophils with red granules. Triangles show tissue macrophages. Right side graphs: Evaluation of pathological changes in the murine airway. All values are expressed as mean ± SE (n = 6). ^*^p < 0.05 vs. WT Control; ^†^p < 0.05 vs. WT PM2.5; ^‡^p < 0.05 vs. WT OVA; ^¶^p < 0.05 vs. WT OVA + PM2.5; ^I^p < 0.05 vs. TLR2^−/−^ Control; ^#^p < 0.05 vs. TLR2^−/−^ PM2.5; ^κ^p < 0.05 vs. TLR2^−/−^ OVA; ^£^p < 0.05 vs. TLR2^−/−^ OVA + PM2.5; ^§^p < 0.05 vs. TLR4^−/−^ Control; ^δ^p < 0.05 vs. TLR4^−/−^ PM2.5; ^&^p < 0.05 vs. TLR4^−/−^ OVA; ^Σ^p < 0.05 vs. TLR4^−/−^ OVA + PM2.5; ^α^p < 0.05 vs. MyD88^−/−^ Control; ^β^p < 0.05 vs. MyD88^−/−^ PM2.5.

No pathologic alterations were found in the lungs of the control group in WT, TLR2−/−, TLR4−/− and MyD88−/− mice (Fig. 3A,D,G,J). PM2.5 alone caused peribronchiolar inflammation in WT mice (Fig. 3B). Infiltration of inflammatory cells (neutrophils and lymphocytes) into the airway submucosa was observed (arrows). However, the degree of peribronchiolar inflammation was very slight in TLR2^−/−^ and TLR4^−/−^ mice (Fig. 3E,H), but no significant changes in the airway of MyD88^−/−^ mice (Fig. 3K).

OVA + PM2.5 caused proliferation of goblet cells in the airway epithelium in WT mice, TLR2^−/−^ and TLR4^−/−^ mice (Fig. 3C,F,I; thin arrows). However, no significant pathological changes in MyD88^−/−^ mice were observed (Fig. 3L). In the pathological scores, the degree of goblet cell proliferation between groups was WT mice > TLR2^−/−^ mice > TLR4^−/−^ mice > MyD88^−/−^ mice (Fig. 3). OVA + PM2.5 caused infiltration of eosinophils, neutrophils, and lymphocytes into the airway submucosa, especially in WT mice (Fig. 3M). However, no significant pathological changes in the lungs of MyD88^−/−^ mice were observed (Fig. 3P). In the pathological scores, the degrees of eosinophiol and lymphocyte infiltration into the airway submucosa between groups were WT mice > TLR2^−/−^ mice > TLR4^−/−^ mice > MyD88^−/−^ mice (Fig. 3). These pathological scores in the OVA + PM2.5 group of WT, TLR2^−/−^, TLR4^−/−^ and MyD88^−/−^ mice were parallel to the induction revels of Th2 cytokines and eosinophil-relevant chemokines in these groups.

### OVA-specific IgE and IgG1 in serum

**Figure 4:**
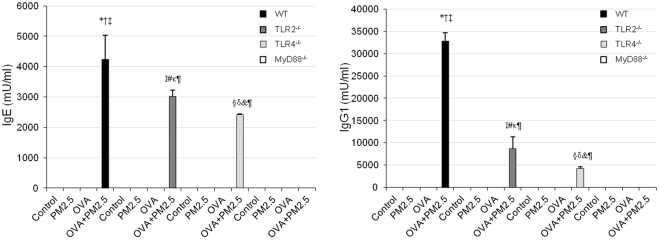
OVA-specific IgE and IgG1 in serum. According to the manufacturer’s protocol, 1 U of anti-OVA IgE is defined as 1.3 ng of the antibody, and 1 U of anti-OVA IgG1 as 160 ng of the antibody. Results are expressed as mean ± SE (n = 6). *p < 0.05 vs. WT Control; ^†^p < 0.05 vs. WT PM2.5; ^‡^p < 0.05 vs. WT OVA; ^¶^p < 0.05 vs. WT OVA + PM2.5; ^I^p < 0.05 vs. TLR2^−/−^ Control; ^#^p < 0.05 vs. TLR2^−/−^ PM2.5; ^κ^p < 0.05 vs. TLR2^−/−^ OVA; ^§^p < 0.05 vs. TLR4^−/−^ Control; ^δ^p < 0.05 vs. TLR4^−/−^ PM2.5; ^&^p < 0.05 vs. TLR4^−/−^ OVA.

OVA alone caused no induction of OVA-specific IgE and IgG1. OVA + PM2.5 significantly increased OVA-specific IgE and IgG1 production in WT mice, TLR2^−/−^ and TLR4^−/−^ mice compared to OVA only-hosts, whereas these antibodies were undetectable in MyD88−/− mice in Fig. 4.

## Discussion

The results of the present study indicate that the activation of TLR signalling by PM2.5, which leads to inflammatory cytokine production, may result in activation of the innate and adaptive immunity related to Th2 responses. The results of the present study demonstrated that exposure to PM2.5 alone increased neutrophil numbers in BALF and also increased chemokine KC levels in WT mice. The human homologue of KC, IL-8, recruits and activates neutrophils^23^. However, these alterations were attenuated although not completely suppressed in TLR2^−/−^, TLR4^−/−^ and MyD88^−/−^ mice. Pathologically, PM2.5 caused slight peribronchiolar inflammation in WT mice, but more subtle pathological changes were observed in the gene-deficient mice, especially in MyD88^−/−^ animals. These data indicate that PM2.5 is capable of activating the innate immune system in a MyD88-dependent manner. These data also suggest that contaminating trace LPS and TLR2 ligands (β-glucan, etc) in PM2.5 are strong candidates for the active agents in neutrophilic lung inflammation caused by PM2.5. A recent study has reported that PM2.5-induced acute lung inflammation in BALB/c mice might be due, in part, to the production of IL-1β. The activation of the TLR4/MyD88 signaling pathway and NOD-like receptor family, pyrin domain-containing 3 (NLRP3) might be involved in the production process^24^. In the present study, PM2.5 increased IL-1β in WT mice, TLR2^−/−^, TLR4^−/−^ and MyD88^−/−^ mice. PM2.5 might cause IL-1β induction through the NLRP3 inflammasome-pathway (MyD88 independent-pathway). It is well known that the inflammasome-pathway is activated by danger signals (peptidoglycan, silica, ATP, uric acid crystal)^25^.

Regarding TLR2 stimuli, hydrogen peroxide (H_2_O_2_), which is generated during inflammation, induces nuclear translocation of NF-κB and AP-1, and phosphorylation of p38 MAPK in neonatal rat ventricular myocytes; anti-TLR2 antibody inhibits these effects. H_2_O_2_ increases NF-κB activation in TLR2-overexpressing Chinese hamster ovary (CHO) fibroblasts, but not in normal or TLR4-overexpressing CHO cells^26^. As metal-containing TLR stimuli, TiO_2_ nanoparticles activate TLR2 and TLR4, and induce expression of TNF-α and NF-κB in mouse hippocampal tissues^27^. Zinc and nickel administration induce inflammatory responses (ICAM-1 and IL-8 induction) in vascular endothelial cells. In these responses, TLR4 plays a dominant role in NF-κB activation by nickel, but not by zinc^28^. In the present study, these metals were included in the tested PM2.5 sample (Ti, 370 ng/mg; Ni, 72 ng/mg). From these reports, we speculate that H_2_O_2_ is generated extracellularly by inflammation, Ti and Ni present in PM2.5 trigger lung inflammation *via* TLRs.

Regarding the relationship between particle size and inflammatory response, overall urban PM2.5-induced inflammatory responses in murine lungs, including those identified in this study, are weaker than those observed in response to coarse PM (PM2.5–PM10μm) or fine particles derived from desert dust (2.5 μm)^15, 16^. We hypothesis that the differences in these inflammatory responses may be due to the differences in amounts of particle-bound microbial elements, especially LPS. However, we currently have reported that type II alveolar cells might react sensitively to oxidative stress induced by PM2.5 and caused an inflammatory response because the response was suppressed by *N*-acetylcystein (anti-oxidants) but not by Polymyxin B (LPS inhibitor)^29^. Therefore, the oxidative stress caused by chemical-rich PM2.5 might play an important role in murine lung inflammation in addition to LPS and metals.

In OVA-treated WT mice, PM2.5 exacerbated OVA-related lung eosinophilia along with an increase in Th2 cytokines (IL-5, and IL-13) and chemokines (eotaxin and MCP-3) and infiltration of eosinophils into the airways. Increasing proliferation of goblet cells in the airway epithelium and production of antigen-specific IgE and IgG1 in serum are exacerbated by allergic inflammatory events. These Th2-derived cytokines and chemokines are key mediators in the symptoms of asthma and are critical for the recruitment and survival of eosinophils^30^, production of antigen-specific antibodies^31^, and the production of mucous cells (e.g., goblet cells) in the bronchial epithelium^32^. All these effects were stronger in TLR2^−/−^ mice than in TLR4^−/−^ mice. In MyD88^−/−^ mice, this pro-inflammatory mediator-inducing ability was very weak and the lung pathology exhibited negligible effect following exposure to the sample mixtures. These results suggest that TLR2 and TLR4 signalling may be important in the exacerbation of PM2.5-induced lung eosinophilia and that MyD88 is a key adapter molecule in this event. Therefore TLR2- and TLR4-ligands, TLR stimuli (H_2_O_2_, metals) and other dinger -stimulus might trigger the exacerbation of lung eosinophilia. However, PM2.5-bound LPS may be a strong candidate for exacerbation of lung eosinophilia caused by PM2.5, indicated by the strong inhibition of effects in TLR4^−/−^ mice.

We have recently reported that antigen-induced allergic inflammation in murine lungs was greater in microbial element (LPS, β-glucan)-rich coarse PM than in organic chemical (PAHs)-rich PM2.5 and have suggested that microbial elements have more potent exacerbating effects on the development of lung eosinophilia than do organic chemicals contained in PM2.5^16^. We have also reported that heated PM2.5-rich dust—heated at 360 °C to exclude toxic materials, such as microbial and chemical elements—caused less effect on neutrophilic lung inflammation and OVA-induced lung esosinophilia in mice than non-heated PM2.5-rich dust^18^. Thus, PM2.5-bound toxic materials are seen to be a key factor for these lung disease enhancements. Therefore, investigation to confirm the phenomenon found by the present study, whether exposure to a mixture of heated PM2.5 and LPS or β-glucan in trace revels causes exacerbation of murine lung eosinophilia or not, is in order.

## Conclusion

This study demonstrates that urban PM2.5 may exacerbate allergic inflammation in the murine lung *via* a TLR2/TLR4/MyD88-signaling pathway. PM2.5-bound trace microbial elements, such as LPS may be a strong candidate for exacerbation of murine lung eosinophilia. The results of the present study suggest that inhalation of urban PM2.5 is a significant risk factor for inflammatory and allergic lung diseases.

## Methods

### Sample collection of PM2.5

PM2.5 samples collected between Jan 20 and Jan 25, 2015 were obtained from China Medial University, Shenyang, China. In China, a massive haze event (density ranges of PM2.5 = 129–291 µg/m^3^) appeared at this time. The experimental air samples were trapped in a four-stage multi-nozzle cascade impactor (MCI) (Tokyo Dylec Co., Tokyo, Japan). An MCI was used to measure the levels of size-classified mass and elemental concentrations of PM2.5 accordance with a previously reported method^15–18^. The MCI consisted of three stages with 12-orifice and back-up stage. The PM2.5 was trapped directly on materials placed behind the jet-nozzles of an MCI set at 20 l/min airflow. The PM2.5 collected was transferred to a sterilized dry bottle, which was placed in a germ free desiccator and stored at −30 °C for further use.

### Particle size

**Figure 5:**
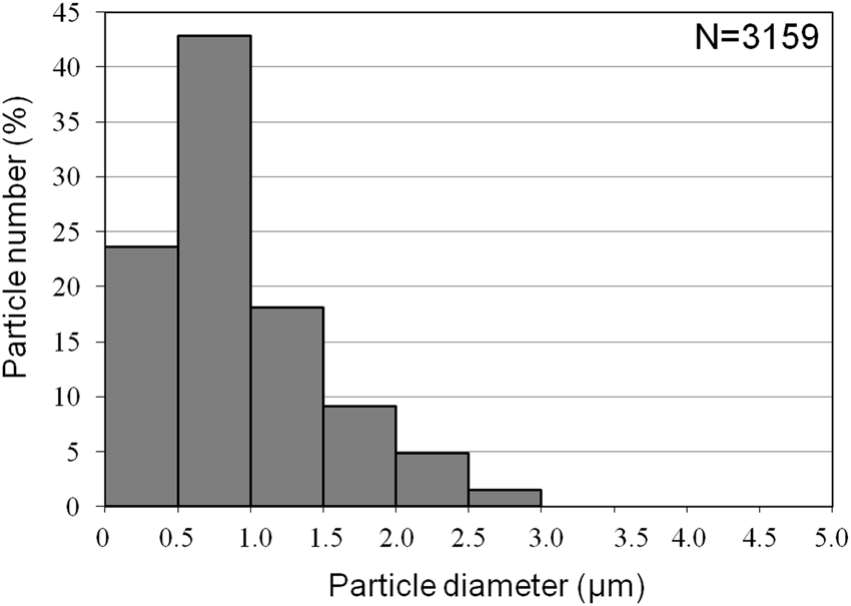
Particle diameter distribution. Particle size was analyzed using a microscope. A total of 3159 particles were measured. Results show the median diameter of PM2.5 to be 0.94 μm. The size distribution peaks of PM2.5 were at 0.5~1.0 μm.

Figure 5 shows the particle size of the PM2.5 analyzed by KEYENCE all-in-one BZ-9000 fluorescence microscope (Osaka, Japan). A total of 3159 particles were counted. The median diameter of the PM2.5 particles was 0.94 ± 0.65 μm (M ± SD). The peak of the size distribution was at 0.5~1.0 μm.

### Analysis of components in the samples

Thermo Scientific model 61E Trace and ICP-750, Thermo Jarrell-Ash inductively-coupled plasma atomic emission spectroscopy (ICP-AES) (Waltham, MA) was used to analyze the elements. Prior to analysis, samples were treated for acid digestion with mixed acids (68% HNO_3_/38% HF/70% HClO_4_ = 5/1/1) for 3 h at 180 °C. An ion chromatograph (DX-100, Dionex, Sunnyvale, CA) and ICP-AES (61E Trace, Thermo Jarrell-Ash) were used to analyze the water soluble components.

### Analysis of microbial elements in particles

Kinetic assays (Seikagaku Corp., Tokyo, Japan) were applied to measure microbial elements: Endospec ES tested MK for LPS activity and Fungitec G tested MK for β-glucan. Briefly, one ml water suspension of PM2.5 (5 mg) was allowed to stand on the bench top at room temperature for 2 h. Subsequently, supernatants recovered were examined for LPS and β-glucan concentrations using Pyro Color-MP Chromogenic Diazo-Coupling Kit (Associates of Cape Cod. Inc., MA, USA).

### Analysis of polycyclic aromatic hydrocarbons (PAHs) in particles

A previously reported method^15–18^ was used to analyze PAHs. Briefly, ultrasonic extraction was performed to extract the air samples trapped on the Teflon filter. The samples were extracted twice with a 20 ml portion of dichloromethane at 15 °C. The filtrate obtained from the extract using a No 5 C filter paper was allowed to stand to dryness in the dark, yielding solid residual substances.

The residual substances dissolved in acetonitrile (0.5 ml) were analyzed for PAHs using a Hitachi Model 600 HPLC (Hitachi, Japan). The HPLC was equipped with a Model L-7485 fluorescence detector (Hitachi, Japan) and a 4.0 mmφ × 250 mm column packed with Wakosil-II 5C 18HG (Waka Pure Chemicals Industry, Ltd., Osaka, Japan). An acetonitrile/water (80/20, v/v) solution was used as a mobile phase at 1.5 ml/min. Identification of unknown PAHs was performed by comparing the HPLC retention time and fluorescence/excitation spectra to those of authentic PAHs^33^ from Supelco (Bellefonte, PA, USA) and Aldrich Chemical Co., Inc. (Milwaukee, WI, USA)/Tridom Chemical Inc (Hauppauge, NY, USA).

### Animals

Homozygous TLR2, TLR4 and MyD88 knockout mice and WT mice (BALB/c parental strain, males) were purchased from Oriental BioService Japan, Inc. (Kyoto, Japan) at 8 weeks of age. Mice were fed with a commercially obtained diet CE-2 (CLEA Japan, Inc., Tokyo) and given water ad libitum. They were placed in plastic cages lined with soft wood chips and kept in a well-controlled room—temperature, 23 °C; humidity, 55–70%, 12 h/12 h light/dark cycle. The conditions used for the present study are in compliance with the U.S. National Institutes of Health Guidelines for the use of experimental animals. The Animal Care and Use Committee at Oita University of Nursing and Health Sciences (Oita, Japan) also approved the method used.

### Study protocol

Four groups (n = 6 per group) of mice (WT, TLR2^−/−^, TLR4^−/−^ and MyD88^−/−^) were prepared for treatments with particles. Each group was treated with sterile saline (control), PM2.5, OVA, and OVA + PM2.5. The suspension of PM2.5 particles for injection was prepared by sonicating a β-glucan (LPS) free sterile saline solution containing 0.9% NaCl (Otsuka Co., Kyoto, Japan) for 5 min with an ultrasonic disrupter, UD-201 type with micro tip (Tomy, Tokyo, Japan) under cooling conditions. Grade VII OVA (Sigma-Aldrich, St. Louis, MO) was dissolved in a sterile saline solution (Otsuka Co., Kyoto, Japan). The instillation volume of the suspension was 80 μl/mouse for diffusing PM2.5 sample into the lungs, 0.1 mg/mouse for PM2.5 and 4 μg per mouse for OVA. Mice treated with a polyethylene tube under 4% halothane anesthesia were intratracheally instilled with a mixed or individual solution of OVA and PM2.5 (Takeda Chemical, Osaka, Japan) 4 times at 2-week intervals. All mice (age = 14 weeks) were anesthetized by intraperitoneal injections of pentobarbital and then euthanized by exsanguination one day after the last intratracheal administration.

### Bronchoalveolar lavage fluid (BALF)

A previously reported method was used to count BALF and cell numbers^15–18, 22^. Briefly, after the collection of blood, tracheas were cannulated. The lungs were lavaged by syringe with two injections of sterile saline (0.8 ml) to lavage the lungs at 37 °C. Gentle aspiration was used to harvest the lavaged fluid. The mean volume retrieved was 90% of the amount instilled (1.6 ml). Fluids from the two lavages were pooled and cooled to 4 °C and then centrifuged at 1500 rpm for 10 min. The protein levels of cytokines and chemokines in the BALF were measured by using the total amount of lavages collected from each individual mouse. A hemocytometer was used to determine the total cell count of fresh fluid specimen. Assessment of differential cell counts was conducted on cytologic preparations. Slides were prepared by Cytospin (Sakura Co., Ltd., Tokyo, Japan) and then stained with Diff-Quik (International Reagents Co., Kobe, Japan). An oil immersion microscopy counted a total of 300 cells. The BALF supernatants were stored at −80 °C until analyzed for cytokines and chemokines.

### Quantitative analysis of cytokines and chemokines in BALF and culture medium

An enzyme-linked immunosorbent assay (ELISA) was used to measure the cytokine protein levels in the BALF. An ELISA kit (R&D Systems Inc., Minneapolis, MN) was used to measure Interleukin (IL)-6, IL-13, eotaxin, keratinocyte chemoattractant (KC), and monocyte chemotactic protein (MCP)-1. Another ELISA kit (Endogen, Cambridge, MA) measured IL-5 and IL-12. MCP-3 was determined using an additional ELISA kit (Bender Med Systems, Burlingame, CA).

### Pathological evaluation

After the BALF cells evaluation, the six mice were pathologically examined. Lungs were treated in a neutral phosphate-buffered formalin solution (10%) and then the lobes were separated (2 mm thick blocks) for paraffin embedding. Embedded blocks sectioned to a 3 μm thickness were stained with May-Grunwald’s stain solution (Nacalai tesque, Inc, Kyoto, Japan) and Giemsa’s azur eosine methylene blue solution (Merck KGaA, Darmstadt, Germany) to evaluate the degree of infiltration of eosinophils or lymphocytes in the airway from proximal to distal. Sections were also stained with periodic acid-Schiff (PAS) (Waka Pure Chemicals Industry, Ltd., Osaka, Japan) for evaluation of the degree of proliferation of goblet cells in the bronchial epithelium. A Nikon ECLIPSE light microscope (Nikon Co, Tokyo, Japan) was used for pathological analysis of the inflammatory cells and epithelial cells in the airway of each lung lobe on the slides. The following scales were used to grade the degree of proliferation of goblet cells in the bronchial epithelium: 0 = not present, 1 = slight, 2 = mild, 3 = moderate, 4 = moderate to marked, and 5 = marked. These words were defined according to percentage of the airway infiltrated with goblet cells stained with PAS: “Slight” = less than 20%, “mild” = 21–40%, “moderate” = 41–60%, “moderate to marked” = 61–80%, and ‘marked’ = more than 81%^16–18^. One May-giemsa or PAS-stained slide per mouse was used to assess pathological changes. Two pathologists, who cross-checked the data with blinded specimens, conducted this evaluation procedure. Values are mean ± SE (n = 6).

### Measurement of Antigen-specific IgE and IgG1 antibodies

A Mouse OVA-IgE ELISA kit and a Mouse OVA-IgG1 ELISA kit (Shibayagi Co., Shibukawa, Japan) were used to measure OVA-specific IgE and IgG1 antibodies, respectively.
